# Supplementary material for: MARCH5 mediates NOXA-dependent MCL1 degradation driven by kinase inhibitors and integrated stress response activation
Source: eLife. 2020 Jun 2;9:e54954. doi: 10.7554/eLife.54954 (PMC7297531; doi:10.7554/eLife.54954)
Supplement: Supplementary file 1. [file elife-54954-supp1.docx]

| **Key Resources Table** | | | | |
| --- | --- | --- | --- | --- |
| **Reagent type (species) or resource** | **Designation** | **Source or reference** | **Identifiers** | **Additional information** |
| strain, strain background (include species and sex here) | DH5α | Invitrogen | 18265-017 | subcloning |
| genetic reagent (human) | HA-tagged MARCH5 plasmid | Sino Biological | HG21559-NY |  |
| cell line (Homo-sapiens) | A549 | PMID: 17145885 | [Kobayashi et al., 2006](https://www.ncbi.nlm.nih.gov/pubmed/17145885) | Kobayashi Lab (BIDMC) |
| cell line (Homo-sapiens) | C4-2 | ATCC | CRL-3314 |  |
| cell line (Homo-sapiens) | DU145 | ATCC | HTB-81 |  |
| cell line (Homo-sapiens) | LNCaP | ATCC | CRL-1740D |  |
| cell line (Homo-sapiens) | HA-tagged MCL1 overexpressing LNCaP | PMID: 30021909 | [Arai et al., 2018](https://www.ncbi.nlm.nih.gov/pubmed/30021909) |  |
| cell line (Homo-sapiens) | MCL1-KO LNCaP | PMID: 30021909 | [Arai et al., 2018](https://www.ncbi.nlm.nih.gov/pubmed/30021909) |  |
| cell line (Homo-sapiens) | MDA-MB-468 | ATCC | HTB-132 |  |
| cell line (Homo-sapiens) | MCF7 | ATCC | HTB-22 |  |
| cell line (Homo-sapiens) | PC3 | ATCC | CRL-1435 |  |
| cell line (Homo-sapiens) | RV1 | ATCC | CRL-2505 |  |
| transfected construct (human) | pooled BIM siRNAs | Dharmacon | L-004383-00-0005 | transfected construct (human) |
| transfected construct (human) | pooled GCN2 (EIF2AK4) siRNAs | Horizon Discovery | L-005314-00-0005 | transfected construct (human) |
| transfected construct (human) | MARCH5 individual siRNA | Thermo Fisher Scientific | s29332 | transfected construct (human) |
| transfected construct (human) | pooled MARCH5 siRNAs | Dharmacon | L-007001-00-0005 | transfected construct (human) |
| transfected construct (human) | pooled MULE (HUWE1) siRNAs | Dharmacon | L-007185-00-0005 | transfected construct (human) |
| transfected construct (human) | pooled NOXA siRNAs | Dharmacon | L-005275-00-0005 | transfected construct (human) |
| transfected construct (human) | three NOXA individual siRNAs | Thermo Fisher Scientific | S10708; S10709; S10710 | transfected construct (human) |
| transfected construct (human) | pooled PERK (EIF2AK3) siRNAs | Horizon Discovery | L-004883-00-0005 | transfected construct (human) |
| transfected construct (human) | control non-target siRNA | Dharmacon | D-001810-01-05 | transfected construct (human) |
| antibody | anti-ATF4 (rabbit monoclonal) | Abcam | ab184909 | Western blot (1:2000) |
| antibody | anti-Bad (rabbit monoclonal) | Cell Signaling Technology | 9239 | Western blot (1:500) |
| antibody | anti-BAK (rabbit monoclonal) | Cell Signaling Technology | 12105 | Western blot (1:1000) |
| antibody | anti-BAX (rabbit monoclonal) | Cell Signaling Technology | 5023 | Western blot (1:1000) |
| antibody | anti-β-Actin (mouse monoclonal) | Abcam | ab6276 | Western blot (1:10000) |
| antibody | anti-BCL2 (rabbit monoclonal) | Cell Signaling Technology | 4223 | Western blot (1:500) |
| antibody | anti-BCLXL (rabbit monoclonal) | Cell Signaling Technology | 2764 | Western blot (1:1000) |
| antibody | anti-BIM (mouse monoclonal) | Santa Cruz Biotechnology | sc-374358 | Western blot (1:500) |
| antibody | anti-BIM (rabbit monoclonal) | Cell Signaling Technology | 2933 | Western blot (1:1000) |
| antibody | anti-cleaved caspase 3 (CC3) | Cell Signaling Technology | 9664 | Western blot (1:250) |
| antibody | anti-FUNDC1 (rabbit polyclonal) | Thermo Fisher Scientific | PA5-48853 | Western blot (1:1000) |
| antibody | anti-HA (rabbit monoclonal) | Cell Signaling Technology | 3724 | Western blot (1:1000) |
| antibody | anti-MARCH5 (rabbit polyclonal) | EMD Millipore | 06-1036 | Western blot (1:2000); Co-IP (5 μg per tube) |
| antibody | anti-MCL1 (mouse monoclonal) | Santa Cruz Biotechnology | sc-12756 | Western blot (1:2000); Co-IP (5 μg per tube) |
| antibody | anti-MCL1 (rabbit monoclonal) | Cell Signaling Technology | 5453 | Western blot (1:1000); Co-IP (5 μg per tube) |
| antibody | anti-Mfn1 (mouse monoclonal) | Santa Cruz Biotechnology | sc-166644 | Western blot (1:1000) |
| antibody | anti-MiD49 (SMCR7) (rabbit polyclonal) | Sigma-Aldrich | SAB2700654 | Western blot (1:1000) |
| antibody | anti-MULE (HUWE1) (mouse monoclonal) | Cell Signaling Technology | 5695 | Western blot (1:500) |
| antibody | anti-NOXA (mouse monoclonal) | Abcam | ab13654 | Western blot (1:250) |
| antibody | anti-p27 (rabbit monoclonal) | Cell Signaling Technology | 3686 | Western blot (1:1000) |
| antibody | anti-p53 (mouse monoclonal) | Santa Cruz Biotechnology | sc-126 | Western blot (1:1000) |
| antibody | anti-p62 (rabbit monoclonal) | Cell Signaling Technology | 5114 | Western blot (1:1000) |
| antibody | anti-PARP (rabbit monoclonal) | Cell Signaling Technology | 9532 | Western blot (1:1000) |
| antibody | anti-phospho-eIF2α Ser51 (rabbit polyclonal) | Cell Signaling Technology | 9721 | Western blot (1:1000) |
| antibody | anti-PUMA (rabbit monoclonal) | Cell Signaling Technology | 12450 | Western blot (1:500) |
| antibody | anti-vinculin (mouse monoclonal) | Santa Cruz Biotechnology | sc-73614 | Western blot (1:20000) |
| recombinant DNA reagent | MARCH5 CRISPR/Cas9 knockout (KO) plasmid | Santa Cruz Biotechnology | sc-404655 |  |
| recombinant DNA reagent | MARCH5 HDR plasmid | Santa Cruz Biotechnology | sc-404655-HDR |  |
| recombinant DNA reagent | Control CRISPR/Cas9 plasmid | Santa Cruz Biotechnology | sc-418922 |  |
| sequence-based reagent | primer mix for *MARCH5* | Thermo Fisher Scientific | Hs00215155_m1 | TaqMan assay |
| sequence-based reagent | primer mix for *MCL1* | Thermo Fisher Scientific | Hs01050896_m1 | TaqMan assay |
| sequence-based reagent | primer mix for NOXA (*PMAIP*) | Thermo Fisher Scientific | Hs00560402_m1 | TaqMan assay |
| sequence-based reagent | primer mix for *18S rRNA* | Thermo Fisher Scientific | 4319413E | TaqMan assay |
| sequence-based reagent | primer mix for *GAPDH* | Thermo Fisher Scientific | 4326317E | TaqMan assay |
| peptide, recombinant protein | epidermal growth factor (EGF) | Sigma-Aldrich | E9644 |  |
| commercial assay or kit | RNeasy Mini kit | Qiagen | 74104 | RNA extraction |
| commercial assay or kit | Mitochondria isolation kit | Thermo Fisher Scientific | 89874 |  |
| chemical compound, drug | ABT-199 | Selleckchem | S8048 |  |
| chemical compound, drug | ABT-263 | Selleckchem | S1001 |  |
| chemical compound, drug | actinonin | Sigma-Aldrich | A6671 |  |
| chemical compound, drug | AZD5991 | AstraZeneca | AZD5991 |  |
| chemical compound, drug | actinomycin D | MedChemExpress | HY-17559 |  |
| chemical compound, drug | cabozantinib | Selleckchem | S1119 |  |
| chemical compound, drug | CPI-613 | MedChemExpress | HY-15453 |  |
| chemical compound, drug | dinaciclib | Selleckchem | S2768 |  |
| chemical compound, drug | erlotinib | Selleckchem | S7786 |  |
| chemical compound, drug | gamitrinib-TPP | PMID: 19229106 | [Kang et al., 2009](https://www.ncbi.nlm.nih.gov/pubmed/19229106?dopt=Abstract) | Altieri Lab (The Wister Institute) |
| chemical compound, drug | ISRIB trans-isomer | MedChemExpress | HY-12495 |  |
| chemical compound, drug | lapatinib | Selleckchem | S2111 |  |
| chemical compound, drug | MG-115 | Sigma-Aldrich | C6706 |  |
| chemical compound, drug | MG-132 | MedChemExpress | HY-13259 |  |
| chemical compound, drug | MLN4924 | MedChemExpress | HY-70062 |  |
| chemical compound, drug | S63845 | MedChemExpress | HY-100741 |  |
| chemical compound, drug | Z-DEVD-FMK | MedChemExpress | HY-12466 |  |
| software, algorithm | R software | R | version 3.3.2 |  |
| other | Seahorse Extracellular Flux analyzer | Agilent Technologies | XFe-96 | mitochondrial function |
| other | SuperSep Phos-tag gel | FUJIFILM WAKO Chemicals | 198-17981 | protein phosphorylation |
